# Improved detection of gene-microbe interactions in the mouse skin microbiota using high-resolution QTL mapping of 16S rRNA transcripts

**DOI:** 10.1186/s40168-017-0275-5

**Published:** 2017-06-06

**Authors:** Meriem Belheouane, Yask Gupta, Sven Künzel, Saleh Ibrahim, John F. Baines

**Affiliations:** 1 Max Planck Institute for Evolutionary Biology, August-Thienemann-Str. 2, 24306 Plön, Germany; 2 Institute for Experimental Medicine, Christian-Albrechts-University of Kiel, Arnold-Heller-Str. 3, 24105 Kiel, Germany; 3 Lübeck Institute of Experimental Dermatology, University of Lübeck, Lübeck, Germany

**Keywords:** QTL mapping, Skin microbiota, 16S rRNA transcript, Skin cancer

## Abstract

**Background:**

Recent studies highlight the utility of quantitative trait locus (QTL) mapping for determining the contribution of host genetics to interindividual variation in the microbiota. We previously demonstrated that similar to the gut microbiota, abundances of bacterial taxa in the skin are significantly influenced by host genetic variation. In this study, we analyzed the skin microbiota of mice from the 15th generation of an advanced intercross line using a novel approach of extending bacterial trait mapping to both the 16S rRNA gene copy (DNA) and transcript (RNA) levels, which reflect relative bacterial cell number and activity, respectively.

**Results:**

Remarkably, the combination of highly recombined individuals and 53,203 informative SNPs allowed the identification of genomic intervals as small as <0.1 megabases containing single genes. Furthermore, the inclusion of 16S rRNA transcript-level mapping dramatically increased the number of significant associations detected, with five versus 21 significant SNP-bacterial trait associations based on DNA- compared to RNA-level profiling, respectively. Importantly, the genomic intervals identified contain many genes involved in skin inflammation and cancer and are further supported by the bacterial traits they influence, which in some cases have known genotoxic or probiotic capabilities.

**Conclusions:**

These results indicate that profiling based on the relative activity levels of bacterial community members greatly enhances the capability of detecting interactions between the host and its associated microbes. Finally, the identification of several genes involved in skin cancer suggests that similar to colon carcinogenesis, the resident microbiota may play a role in skin cancer susceptibility and its potential prevention and/or treatment.

**Electronic supplementary material:**

The online version of this article (doi:10.1186/s40168-017-0275-5) contains supplementary material, which is available to authorized users.

## Background

Mammals host a wide range of complex and diverse microbial communities in association with their barrier organs, which contribute to critical aspects of host biology. Accordingly, many studies of the resident microbiota in the past decade provided primary surveys in a variety of different contexts (e.g., organ, disease state, genotype, development, geography) [1–5]. A salient feature of these studies is the revealing of substantial diversity between individuals. Thus, a thorough understanding of the fundamental factors that govern the assembly and stability of bacterial communities is of critical importance. Broadly speaking, studies addressing these questions identify the environment, diet, and host genetics as important contributors to interindividual variation in host-associated communities [6–8].

The influence of host genetics on bacterial community structure has been addressed by a number of different approaches in human and mouse models, such as twin studies [9, 10], comparison of mouse inbred strains [11, 12], quantitative trait locus (QTL) analysis [13, 14], and more recently first genome-wide association studies [15–19]. These studies provide valuable insights into the role of host genetics in shaping the structure of the bacterial communities, although are largely limited to gut communities.

The skin offers numerous niches for microbes and accordingly harbors complex bacterial communities [20], which differ between distinct body sites and individuals. However, individuals display greater intrapersonal than interpersonal similarity within a specific skin habitat over time [21, 22], suggesting that in addition to other principles governing community stability, host genetics may be a contributing factor to these observed patterns. In a recent study, we addressed whether host genetic variation contributes to variation in the skin microbiota in mice by employing a QTL mapping approach to the fourth generation of an advanced intercross line (AIL) [23]. This revealed a total of 13 regions of the mouse genome significantly associated to skin bacterial traits, although the large size of the defined genomic regions (from 9 to 33 megabases) did not easily allow for more detailed characterization of the individual genes involved.

In this study, we aimed to improve the mapping resolution of QTLs for skin microbial abundances by using the 15th generation of the previously used AIL [23], which we generated through the continuous random intercrossing of individuals, and by increasing the marker density to 53,203 informative SNPs. Furthermore, we introduced a novel means of microbial phenotyping in a QTL context by performing 16S ribosomal RNA (rRNA) profiling at both the gene copy (DNA) and transcript (RNA) levels, which reflect relative bacterial cell number and activity, respectively. Our analysis reveals numerous genomic regions associated with skin microbial abundances, in several cases containing single immune- and/or skin cancer-related genes, whereby 16S rRNA transcript-level profiling was considerably more effective in terms of the number of associations identified. This suggests that bacterial activity levels may provide deeper insight into mechanisms of host-microbe interactions than DNA-level profiling alone.

## Results

### Skin microbiota composition in the AIL mapping population

In order to fine-map genomic regions influencing bacterial taxon abundances in the skin, we analyzed a total of 270 mice from the 15th generation (hereafter G_15_) of the same AIL from which we previously analyzed the 4th generation (G_4_) [23]. An important factor to consider when analyzing the G_15_ mice is that the AIL was transferred from an animal facility at the University of Rostock to a new animal facility at the University of Lübeck, shortly after the analysis of the G_4_ mice was conducted. Thus, prior to QTL mapping, we conducted a thorough ecological analysis of the G_15_ mice in order to re-evaluate the bacterial taxa present and to aid the interpretation of mapping results. Furthermore, given that the skin harbors comparatively low biomass communities and is in constant contact with the environment, we reasoned that bacterial 16S rRNA transcripts may in some cases better reflect true resident skin bacteria interacting with their host. Thus, we performed 16S rRNA gene amplicon (V1–V2 hypervariable regions) sequencing on the Illumina MiSeq platform using both bacterial genomic DNA and RNA reverse transcribed into complementary DNA (cDNA) as template. In total, we analyzed nearly two million sequences after quality filtering and processing, with a normalized coverage of 3500 sequences per sample for each of the DNA- and RNA-based datasets, which we refer to as the “standing” and “active” communities, respectively.

**Fig. 1:**
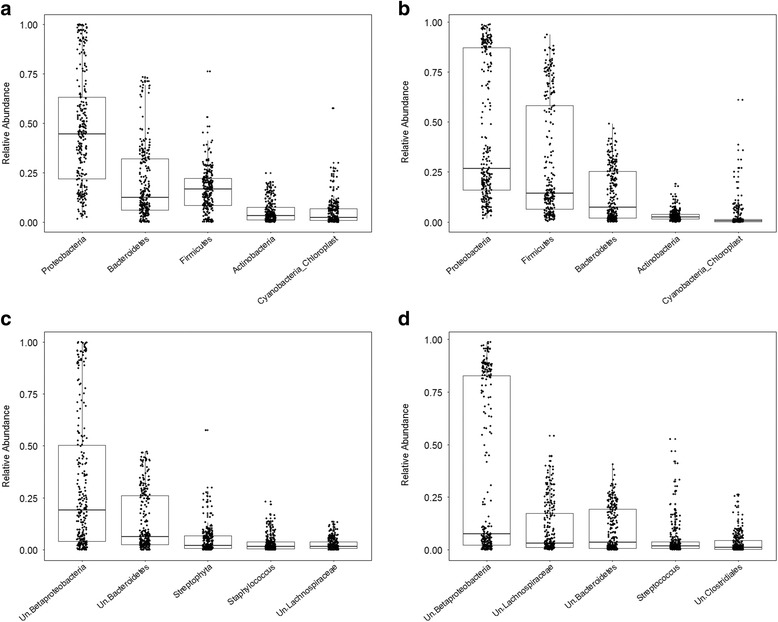
Relative abundances of phyla and genera. The five most abundant phyla and genera are shown. **a** Major phyla in standing (DNA-based) communities. **b** Major phyla in active (RNA-based) communities. **c** Major genera in standing (DNA-based) communities. **d** Major genera in active (RNA-based) communities. *Un* unclassified

First, we analyzed community composition at the phylum and genus levels (Fig. 1). Overall, the mean relative abundances of the major phyla and genera vary largely between the standing and active datasets (Table 1). Proteobacteria is the most abundant phylum in both standing and active datasets (46 and 44%, respectively) and does not significantly differ between them. Bacteroidetes and Firmicutes, on the other hand, display significant contrasting patterns between the standing and active communities, with Bacteroidetes being more abundant at the DNA compared to the RNA level (19 versus 13%, respectively) and Firmicutes being almost twofold more abundant at the RNA compared to the DNA level (29 versus 16%, respectively). Actinobacteria and Cyanobacteria make up a smaller proportion of the standing communities (5.3 and 4.7%, respectively) and are further significantly reduced in the active communities (3 and 2.5%, respectively). These relative patterns at the RNA compared to the DNA level are also largely reflected by the respective most abundant genera belonging to each of these phyla (Fig. 1c, d; Table 1).

**Table 1:** Mean relative abundances of major taxa between standing and active communities in the G_15_ population

Rank	Taxon	Paired Wilcoxon test
Phylum	Proteobacteria	0.43
Phylum	Bacteroidetes	*1.68 × 10* ^*−12*^
Phylum	Firmicutes	*3.97 × 10* ^*−11*^
Phylum	Actinobacteria	*2.24 × 10* ^*−5*^
Phylum	Cyanobacteria_Chloroplast	*3.97 × 10* ^*−11*^
Genus	*Streptophyta*	*6.65 × 10* ^*−10*^
Genus	*Staphylococcus*	*6.04 × 10* ^*−10*^
Genus	Un.Lachnospiraceae	*2.2 × 10* ^*−15*^
Genus	*Streptococcus*	*6.65 × 10* ^*−10*^
Genus	Un.Clostridiales	*1.5 × 10* ^*−5*^

Significant *p* values (≤0.05) after Benjamini-Hochberg [27] correction for multiple testing are indicated in italics

*Un* unclassified

**Fig. 2:**
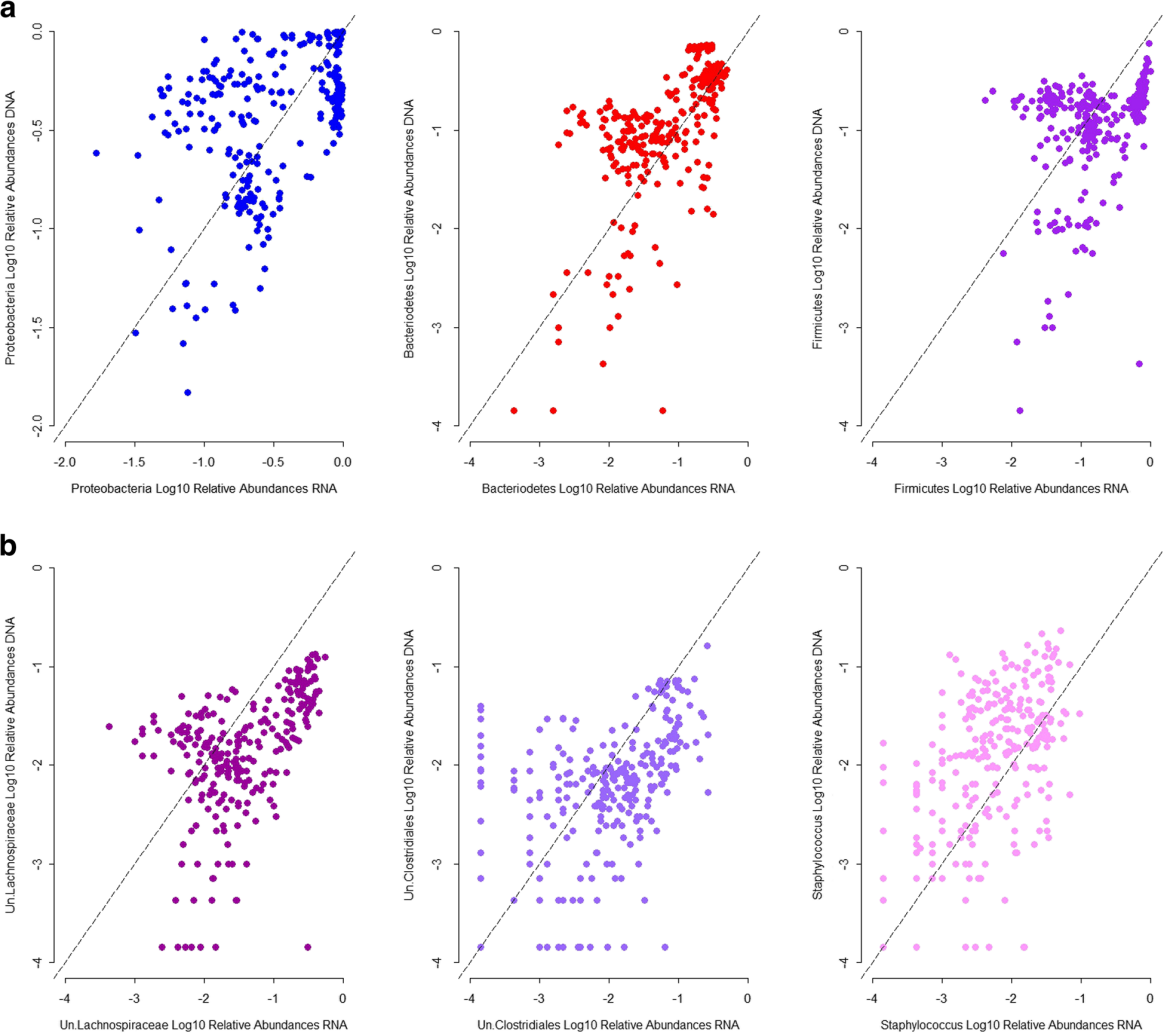
Correlation between standing and active relative abundances for representative taxa. **a** Phyla. **b** Genera. Spearman’s correlation: Proteobacteria: *r* = 0.42, *p* = 4.19 × 10^−13^; Bacteroidetes: *r* = 0.66, *p* = 3.3 × 10^−16^; Firmicutes: *r* = 0.39, *p* = 2.72 × 10^−11^; Un.Lachnospiraceae: *r* = 0.56, *p* = 3.3 × 10^−16^; Un.Clostridiales: *r* = 0.50, *p* = 3.3 × 10^−16^; *Staphylococcus*: *r* = 0.48, *p* = 3.3 × 10^−16^. *Un* unclassified. *p* values are adjusted following Benjamini and Hochberg method [27]

To further examine the correspondence of abundances at the DNA and RNA levels for a single taxon, we investigated their correlations. Among the most abundant taxa, we observe overall moderate to poor correlations; Proteobacteria, Bacteroidetes, unclassified Lachnospiraceae, unclassified Clostridiales, and *Staphylococcus* abundances show a moderate, positive, and significant correlation, whereas Firmicutes abundances correlate poorly between the standing and active datasets (Fig. 2a, b). This indicates that the presence and activity of taxa vary distinctively across individuals and bacterial groups.

**Fig. 3:**
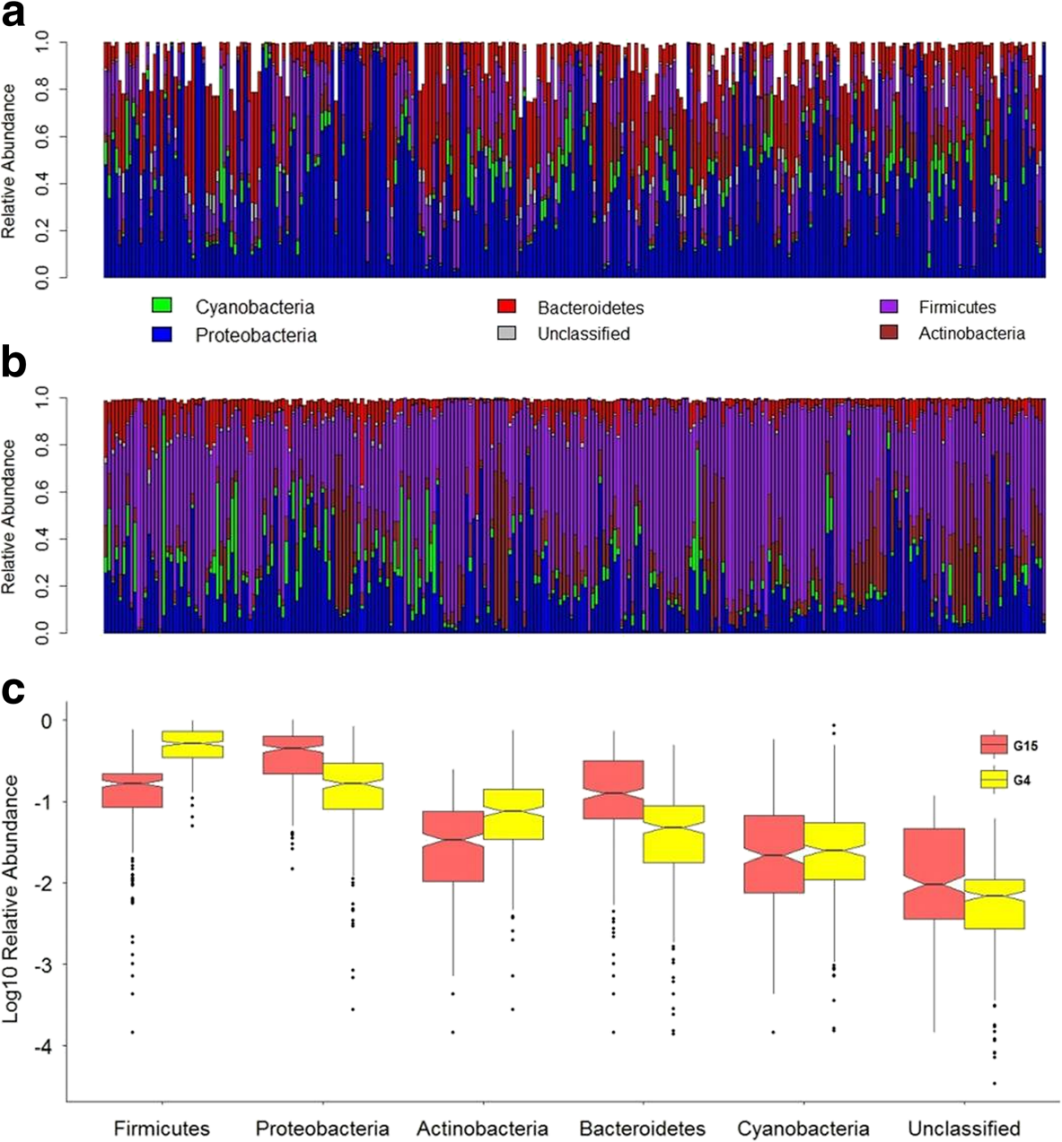
Comparison of skin microbiota composition between G_4_ and G_15_ populations. **a** Bar plot of phylum abundances in the G_15_ population. **b** Bar plot of phylum abundances in the G_4_ population. **c** Boxplots of log_10_-transformed mean relative abundances of major phyla in populations G_4_ and G_15_. ANOVA: Firmicutes, *p* = 2.2 × 10^−16^; Proteobacetria, *p* = 2.2 × 10^−16^

Next, we compared the overall community composition between the G_15_ and G_4_ populations. Although there is a large degree of overlap in terms of the major taxa present, significant differences between these two mouse cohorts are already apparent among phylum-level abundances, whereby the former G_4_ cohort is dominated by Firmicutes in contrast to the G_15_, which is dominated by Proteobacteria (Fig. 3a–c). Systematic community-level differences are also clearly revealed by beta diversity analyses (Additional file 1), whereby the standing and active communities of the G_15_ display much more similarity to each other than either does to the standing communities of the G_4_, despite the differences in abundance between the DNA- and RNA-based profiling outlined above.

### Patterns of variation among Core Measurable Microbiota taxa

For further analysis, we defined a “Core Measurable Microbiota” (CMM) [13] (see “Methods”) for the G_15_ population, which in total contains 92 taxa from the genus to the phylum level and 44 species-level operational taxonomic units (OTUs). The CMM traits represent a small fraction (9 and 0.2%, for CMM taxa and OTUs, respectively) of all detected taxa, but their cumulative abundances represent more than 90 and 60% of taxon and OTU abundances, respectively, within the entire standing and active datasets. Due to the known technical challenges of metagenomic analysis of low microbial biomass samples such as the skin [24, 25], we evaluated the possible influence of contamination during experimental procedures on our ability to reliably measure the CMM and other traits included in the QTL analysis (CMM traits plus those previously significant in the G_4_ population, see below) using “SourceTracker” [26]. This analysis reveals minimal estimates of contamination for the mapped genera and species-level OTUs (97% similarity threshold), whereby estimates for the active communities are in each case lower (mean ± standard deviation, genus: DNA 5.2 ± 3.4%, RNA 3.8 ± 3.5%; OTU: DNA 3.2 ± 2.2%, RNA 2.5 ± 2.5%).

To assess the degree of interindividual variation within each CMM trait, we calculated summary statistics of the relative abundances in the standing and active datasets (Additional files 2, 3, 4, and 5). The relative abundances of the CMMs vary greatly across individuals. For example, in the standing communities, unclassified Betaproteobacteria ranges from 1.5 to 100%, while *Staphylococcus_OTU16* varies from 1.3 to 17.3%. Examples in the active communities include *Campylobacter*, which ranges from 2.1 to 31.9%, and *Acinetobacter_OTU18*, which ranges from 0.7 to 44.9%. As expected, the most abundant CMMs harbor the highest dispersion across individuals, whereas the least abundant ones display tighter dispersion.

To measure the influence of the cage environment, gender, and age on the interindividual variation in the CMMs, we built a mixed effects model for each CMM trait using the log_10_-transformed relative abundance as the response variable, gender and age as fixed explanatory variables, and cage as a random term, separately for the standing and active communities. Accordingly, we quantified the fractions of total variance explained by each of these factors (Additional files 6, 7, 8, and 9), which varies considerably across CMM traits and between the standing and active datasets. For example, in the standing communities, only 0.52% of the total variance in Enterobacteriaceae abundance is explained by cage, whereas cage explains 32.98% of the total variance in Deltaproteobacteria abundance, and similar patterns are observed for the active communities. Within individual CMM traits, some display large correspondence between the standing and active communities (e.g., cage explains 21.14 and 17.20% of variation in Peptostreptococcaceae abundance in DNA- and RNA-based data, respectively), whereas others do not (e.g., cage explains 12.05 compared to 0% of the variation in *Alistipes* abundance in DNA- compared to RNA-based data, respectively). On average, the fraction of total variance explained by cage is higher in the standing compared to active communities (DNA: genus to phylum taxa 12.91%, species 12.67%; RNA: genus to phylum taxa 10.58%, species 9.42%). Similar to the cage environment, the variance explained by gender and age also fluctuates substantially across CMM traits and their relative patterns in the standing and active communities. However, the fraction of total variance explained by gender and age combined is higher in the active compared to standing communities (DNA: genus to phylum taxa 12.44%, species 12.59%; RNA: genus to phylum taxa 25.26%, species 16.61%). Importantly, after accounting for cage, gender, and age effects, the remaining residual variation still comprises the greatest proportion of total variance for nearly all CMM traits. The residuals for all mapped traits are provided in Additional file 10.

### QTL mapping of the skin microbiota in the G_15_

**Fig. 4:**
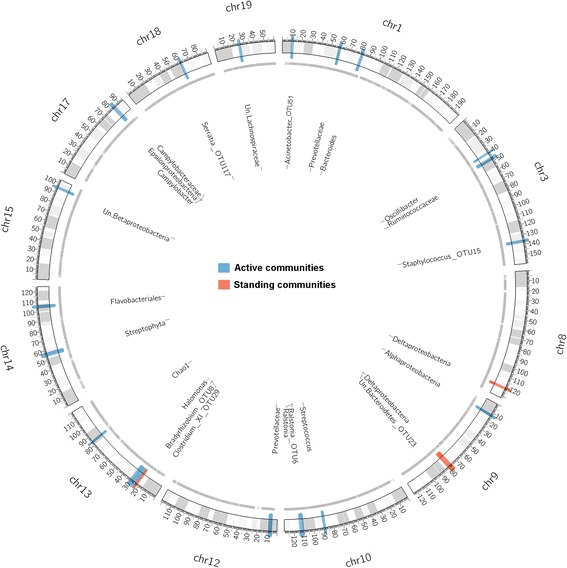
QTL mapping of the standing and active microbiota in the G_15_ population. Only chromosomes with identified QTLs are shown. *Black lines* on the chromosomes denote SNPs used in the mapping, and each *colored region* denotes a QTL defined on either the standing (DNA) or active (RNA) communities

To identify regions of the host genome influencing variation in skin microbial traits in the G_15_ population, we performed linkage mapping (see “Methods”) on the 136 CMM traits described in addition to alpha diversity. Further, in an attempt to potentially replicate previously identified QTLs, we additionally included those CMM traits that showed significant associations with the host genome in the G_4_ and are present in the G_15_, but do not meet the criteria to be defined as part of the CMM in the G_15_. In total, we identified 13 significant (*p* ≤ 0.05) and 12 suggestive (*p* ≤ 0.1) QTLs among the standing and active community traits (Fig. 4, Table 2). Notably, QTL sizes span narrow confidence intervals ranging from 5 to 0.08 Mb, which in some cases contain single genes, and the phenotypic variance explained by individual peak SNPs averaged across all traits accounts for approximately 9% of the total variance. Five QTLs are defined for the standing communities, two of which have pleiotropic effects. For example, the genomic region on chromosome 9, ranging from 77 to 80 Mb, is associated with variation in both Deltaproteobacteria and Bacteroidetes*_OTU23.* In comparison, 21 QTLs are present among the active communities, none of which overlap with those identified for the standing communities. Two of the active QTLs are for Prevotellaceae, whereas the same region is identified at the genus and species levels for *Ralstonia* (Table 2). Further, we identified a single QTL influencing genus-level alpha diversity (Chao1) in the active communities.

**Table 2:** QTL statistics of the standing and active CMM traits in the G_15_ population

	Trait	Category	Chr	Peak SNP	Position	LOD	CI (Mb)	Phenotypic variance	Size	Region defined in G_4_	G_4_ CI (Mb)	G_4_ trait
DNA	Deltaproteobacteria	Order	8	UNC15609494	121.36	*6.21*	121.24–121.54	10.05	0.303	No	–	
	Deltaproteobacteria	Order	9	UNC16678197	77.29	5.26	77.10–77.69	8.59	0.594	No	–	
	Un.Bacteroidetes_OTU23	Species	9	JAX00699225	77.88	5.34	77.22–80.12	8.7	2.9	No	–	
	*Bradyrhizobium_OTU8*	Species	13	UNC22265895	23.92	*5.6*	23.17–23.93	9.11	0.759	No	–	
	*Clostridium_XI_OTU29*	Species	13	UNC22262889	23.69	5.29	23.15–24.79	8.64	1.64	No	–	
RNA	*Acinetobacter_OTU51*	Species	1	JAX00241740	10.25	*7.47*	9.95–10.26	11.97	0.315	No	–	
	Prevotellaceae	Family	1	backupUNC010101265	58.43	5.34	58.19–58.49	8.7	0.294	No	–	
	*Bacteroides*	Genus	1	JAX00257146	81.95	5.09	81.30–81.95	8.32	0.648	No	–	
	*Oscillibacter*	Genus	3	UNC5110176	40.79	*5.46*	40.69–40.80	8.9	0.115	No	–	
	Ruminococcaceae	Family	3	UNC030426787	47.47	5.2	46.61–47.74	8.48	1.136	Yes	46–55	Flavobacteria Cyanobacteria
	*Staphylococcus_OTU15*	Species	3	UNC6336040	136.88	*5.79*	136.35–136.88	9.41	0.528	No	–	
	Alphaproteobacteria	Order	9	JAX00168342	13.14	*5.73*	12.78–13.39	9.31	0.602	No	–	
	*Streptococcus*	Genus	10	JAX00295481	89.09	5.15	89.08–89.23	8.4	0.147	No	–	
	*Ralstonia*	Genus	10	UNC18732289	111.75	5.29	111.57–113.68	8.63	2.116	No	–	
	*Ralstonia_OTU6*	Species	10	UNC18732289	111.75	*5.76*	111.57–112.19	9.36	0.62	No	–	
	Prevotellaceae	Family	12	JAX00324994	8.05	5.56	6.59–8.05	9.05	1.453	No	–	
	*Halomonas*	Genus	13	JAX00042892	27.28	*5.75*	25.17–30.24	9.34	5.077	No	–	
	Chao1	Diversity	13	UNC22977994	84.02	*5.57*	84.00–84.18	9.06	0.187	No	–	
	*Streptophyta*	Genus	14	JAX00380739	57	*5.78*	55.05–57.02	9.39	1.965	Yes	56–69	*Neisseria*
	Flavobacteriales	Class	14	UNC24657914	104.81	5.26	104.71–105.39	8.59	0.678	No	–	
	Un.Betaproteobacteria	Genus	15	UNC26160173	95.24	5.24	95.01–95.39	8.55	0.379	Yes	82–101	*Herbaspirillum*
	Epsilonproteobacteria	Order	17	UNC28472672	83.04	*5.83*	82.58–83.62	9.47	1.05	Yes	76–95	Enterobacteriaceae
	Campylobacteraceae	Family	17	UNC28472672	83.04	5.12	82.58–83.62	8.36	1.05	Yes	76–95	Enterobacteriaceae
	*Campylobacter*	Genus	17	UNC28493644	84.45	**5.3**	84.39–84.47	8.65	0.083	Yes	76–95	Enterobacteriaceae
	*Serratia_OTU117*	Species	18	UNC29400134	61.7	*9.41*	61.50–61.73	14.83	0.24	No	–	
	Un.Lachnospiraceae_OTU82	Species	19	UNC30107716	25.2	*5.78*	25.15–25.35	9.39	0.194	No	–	

LOD scores in italics indicate significance at *p* ≤ 0.05; peak SNP positions and confidence intervals are given in Mb (from NCBI 37). Phenotypic variance is shown in percent contribution (%) to the total variance of each trait. QTL size is indicated in Mb, and previously defined QTLs in the G_4_ population are shown

*Chr* chromosome, *Un* unclassified, *CI* confidence interval

To further evaluate the reliability of bacterial traits as measured by NGS-based methods, we independently analyzed three bacterial traits for which QTLs were detected (Betaproteobacteria, Epsilonproteobacteria, and *Streptococcus*) by performing qPCR measurements on a random subset of 80 mice using group-specific primers. The relative abundances assessed by sequence profiles and qPCR estimates display significant positive correlations for all three traits (Spearman’s correlation: Epsilonproteobacteria: *r* = 0.40, *p* = 0.0003; unclassified Betaproteobacteria: *r* = 0.25, *p* = 0.02; *Streptococcus*: *r* = 0.36, *p* = 0.0012; *p* values corrected according to Benjamini-Hochberg [27]), thus supporting the reliability of our bacterial phenotyping methods.

To determine whether we replicate previously detected QTLs in the G_4_ population, we compared the identified genomic regions in the G_15_ to our previous study [23]. The most promising trait is *Neisseria*, which was associated to chromosome 14 (confidence interval 56 to 69 Mb) in the G_4_ population. In G_15_, the confidence interval ranges from 60.55 to 60.98 Mb, the LOD score of the peak SNP is 9.01, and the percent explained variance is 14.24%, although it is not significant at our determined genome-wide thresholds (Additional file 11). We did, however, discover four genomic regions contained within regions previously detected in the G_4_ that are significantly associated with four CMM traits in the G_15_ population (Table 2), although the bacterial taxa are not the same.

In addition, we compared the intervals detected in our G_15_ analysis to published human GWAS and mouse QTL studies. While none of the loci previously associated with skin microbial traits in humans [16, 28] were contained within our G_15_ intervals, we detect some overlap with QTL studies of the gut microbiota in mice [3, 13]. These include the QTL on chromosome 10 (111.57–113.68 Mb) for the genus *Ralstonia* and corresponding OTUs, which overlaps with a pleiotropic genomic region from Benson et al. [13] on chromosome 10 associated with Coriobacteriaceae (106–122 Mb) and *Lactococcus* and Streptococcaceae (100–111 Mb). Additionally, our QTL on chromosome 12 (6.59–8.05 Mb) for Prevotellaceae overlaps with a QTL on chromosome 12 (−26 Mb) for Ruminococcaceae identified by Benson et al. [13]. Finally, our QTL located on chromosome 15 (95.01–95.39 Mb) for unclassified Betaproteobacteria overlaps with a QTL for Rikenellaceae on chromosome 15 (92.73–97.39 Mb) identified by McKnite et al. [3].

### Analysis of candidate regions

Due to the narrow confidence intervals identified in the G_15_, we were able to identify many promising candidate genes. In Table 3, we list the genes related to the immune response and/or other skin biological processes contained within our confidence intervals whose functions are supported by experimental evidence. The functions of the potential candidate genes are further summarized in Additional file 12 and can largely be divided into five diverse groups: immune response, interaction with bacteria and viruses, skin developmental processes, susceptibility to autoimmune diseases, and susceptibility to skin cancer. Genes belonging to the latter two groups are the most frequent. Regarding skin cancer, we find genes involved in squamous cell carcinoma (SCC), melanoma, actinic keratosis, skin hyperpigmentation, and epithelial dysplasia. For autoimmune diseases, we report genes mainly associated with psoriasis, inflammatory arthritis, acute allergic reaction, and ichthyosis.

**Table 3:** List of potential candidate genes located in the defined confidence intervals

	Trait	Category	Chr	Immune-related genes	Genes related to skin biological processes
DNA	Deltaproteobacteria	Order	8	–	***Cdh13***
	Deltaproteobacteria	Order	9	*Gclc*	–
	Un.Bacteroidetes_OTU23	Species	9	*Gclc, Fbxo9*, *Ick*, *Gsta4, Eef1a1*	*Gsta4*, *Gsta1*, *Gsta2*, *Cd109*, *Ddx43*,*Col12a1*
	*Bradyrhizobium_OTU8*	Species	13	*Trim38*, *Hfe, Btn1a1*, *Btn2a2*	–
	*Clostridium_XI_OTU29*	Species	13	*Trim38*, *Hfe*, *Btn1a1*, *Btn2a2*, *Cmah*	*Cmah*
RNA	*Acinetobacter_OTU51*	Species	1	*Cops5*	*Arfgef1*
	Prevotellaceae	Family	1	*ClK1*	–
	*Staphylococcus_OTU15*	Species	3	*Ppp3ca*	–
	*Streptococcus*	Genus	10	*Scyl2*	*Scyl2*
	Prevotellaceae	Family	12	–	*Apob*
	*Halomonas*	Genus	13	*Sox4*	*Prl*, *Sox4*, *Cdkal*, *E2f3*
	*Streptophyta*	Genus	14	*Mmp14*, *Psmb11*, *Cebpe*, *Il25*, *Psme1*, *Psme2, Irf9*, *Rnf31*, *Mdp1*, *Rabggta*, *Ripk3*, *Cma1, Mcpt1*, *Mcpt4*, *Mcpt8*, *Ctsg*, *Gzmd, Gzmn, Gzmf*, *Gzmc*, *Gmzb*, *Homez*	*Mmp14*, *Ltb4r2*, *Ltb4r1*, *Psme1*, *Mcpt1, Mcpt4*, *Psme2*, *Prmt5*, *Slc7a8*, *Cmtm5*, *Pck2, Rec8*, *Nedd8*, *Tgm1*, *Dhrs1*, *Ctsg*, *Gzme, Gmzb*, ***Atp12a***, *Tinf2*
	Flavobacteriales	Order	14	–	*Pou4f1*
	Un.Betaproteobacteria	Genus	15	–	***Nell2***
	Epsilonproteobacteria, Campylobacteraceae	Order, family	17	–	*Pkdcc*
	*Serratia_OTU117*	Species	18	–	*Ppargc1b, Csnk1a1*
	Un.Lachnospiraceae_OTU82	Species	19	*Dock8*	*Dock8*

Only genes whose functions are experimentally demonstrated to be related to immune response and/or to other skin biological processes are reported. Genes in bold indicate the presence of the peak SNP

*Chr* chromosome, *Un* unclassified

The significance of the biological functions listed above is also supported by pathway and gene ontology enrichment analyses, which in addition reveal further interesting functions (Additional files 13 and 14). Among the enriched pathways are several involved in apoptosis and tissue homeostasis including apoptotic DNA fragmentation and tissue homeostasis, granzyme A-mediated apoptosis, caspase cascade in apoptosis, and the effects of calcineurin in keratinocyte differentiation. Additional significantly enriched pathways include glutamate metabolism activities, which are also revealed by the biological process analysis. Interestingly, glutamate is a key neurotransmitter of the central nervous system, but recent studies show that glutamate receptors are expressed in non-neuronal tissues such as the skin and that glutamate signaling is dysregulated in numerous cancer forms including melanoma [29, 30]. Further pathways include immune-related functions (e.g., B cell receptor signaling pathway, fMLP-induced chemokine gene expression in HMC-1 cells, and MEF2D in T cell apoptosis) and, interestingly, the nitric oxide signaling pathway. Of note, nitric oxide plays diverse biological functions, and in the skin, it is involved in the maintenance of barrier function, melanogenesis, erythema, immunosuppression, and the protection of keratinocytes against UV-induced apoptosis [31, 32]. On the other hand, the biological process analysis reveals phosphate ion metabolism as the most enriched term. Previous studies show that phosphate, a critical element for dividing cells, likely modulates the activity of cancer cells [33], and elevated levels of serum phosphate have been related to lung and skin carcinogeneses in mouse models [34]. Additional enriched terms include regulation of cell polarity and several transport functions (e.g., anion, cation, sodium, and acidic amino transport).

**Fig. 5:**
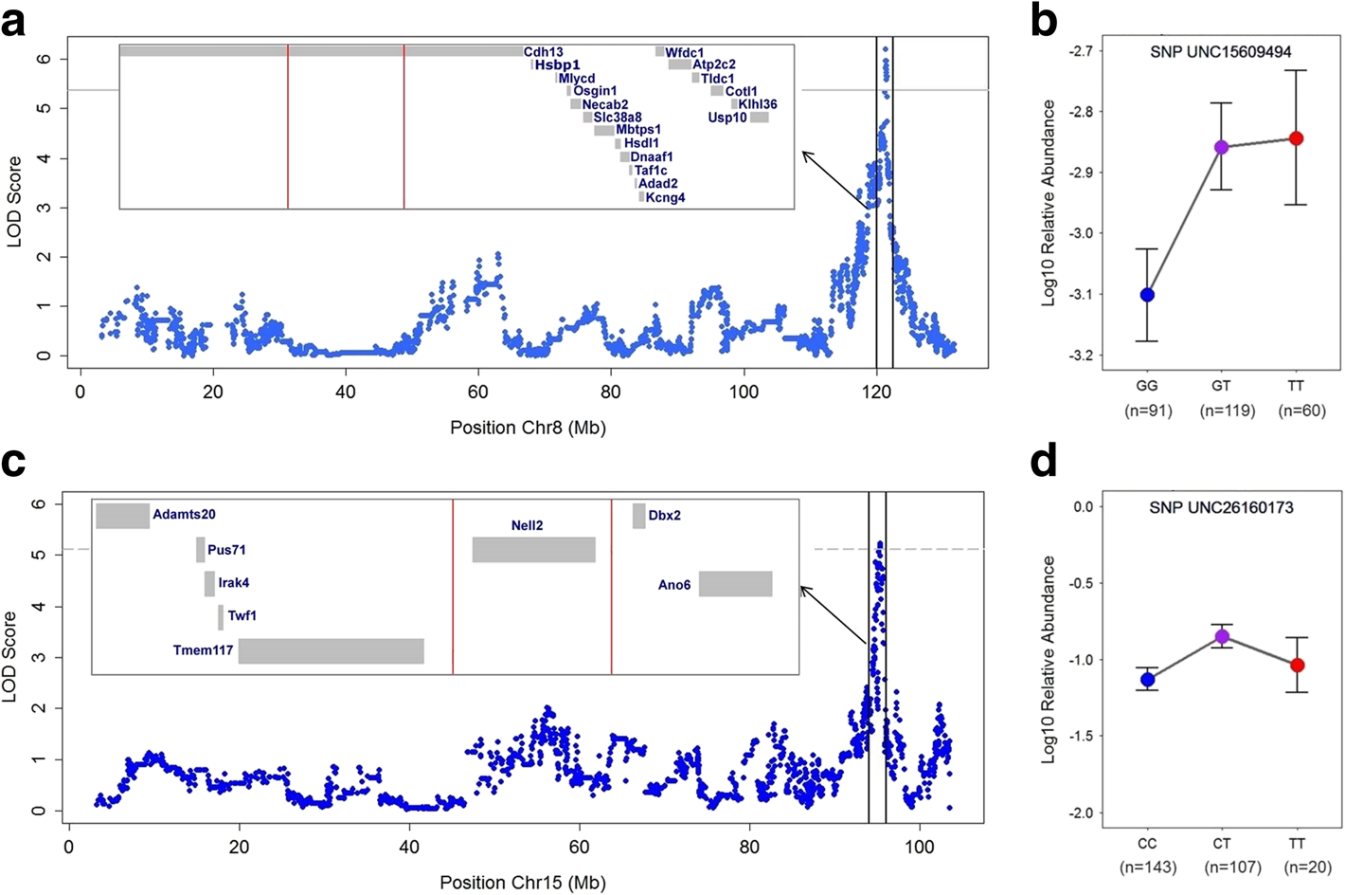
Candidate regions for Deltaproteobacteria and unclassified Betaproteobacteria traits. **a**, **c** Manhattan plots and confidence intervals (*red bars*) for Deltaproteobacteria and unclassified Betaproteobacteria QTL mapping, respectively. **b**, **d** Peak-SNP effect and frequency in QTLs for Deltaproteobacteria and unclassified Betaproteobacteria, respectively. *Bars* represent SE of the mean. *Un* unclassified

Two exceptional candidate regions are those associated with Deltaproteobacteria (standing communities) and unclassified Betaproteobacteria (active communities), which each contain only a single gene: cadherin 13 (*Cdh13*) and neural epidermal growth factor-like 2 (*Nell2*), respectively (Fig. 5). *Cdh13* codes for a cell adhesion molecule that is specifically expressed in the basal keratinocytes of the human and mouse epidermis [35]. *Cdh13* expression is also observed to be significantly reduced in both invasive cutaneous SCC and psoriasis vulgaris lesions and is thus considered to be an endogenous negative regulator of keratinocyte proliferation and also a crucial preserver of healthy skin architecture [36–38]. *Nell2*, on the other hand, is reported to be specifically expressed in the epidermis (keratinocytes) of patients suffering from atopic dermatitis [39]. Several other noteworthy candidates are described in the “Discussion.”

## Discussion

In this study, we performed the first high-resolution genetic mapping of skin microbial traits in the mouse genome. While the overall degree of replication between the G_4_ and G_15_ is low (possible reasons are discussed below), up to five genomic regions display some evidence of overlap between the two cohorts, and the greatly improved results of the G_15_ analysis provide several points of novel insight into the nature of host-microbe interactions in the skin. This is on the one hand made possible by the highly advanced nature of the mapping population (i.e., the 15th generation of an advanced intercross) and high marker density (>50,000 informative SNPs). On the other hand, we also introduced a novel means of microbial “phenotyping” in a QTL setting by performing 16S rRNA profiling on the transcript level, which proved to be more effective in detecting significant associations. One of the most surprising and intriguing aspects of our findings is the preponderance of identified candidate genes involved in cancer-related processes, which is also supported by aspects of the microbial traits themselves as discussed below.

### Comparison between the G_4_ and G_15_ populations

Many potential factors could contribute to the overall lack of replication we observe between the G_4_ and G_15_ study. From the genetic perspective, Greene et al. [40], for example, addressed the question of failing to replicate genetic associations between distinct datasets and concluded (using simulations) that in part, changes in allele frequency can lead to opposite allelic effects on the phenotype. Further, a recent QTL study of the gut microbiota performed on two distinct generations of the same advanced intercross line (generations 4 and 10) similarly replicated and refined only four genomic regions in the more advanced generation, whereby three of the QTLs concerned phylogenetically related traits between cohorts. The authors explained that this poor replication could be due to false-positive QTLs and/or a disparate microbiome composition between the generations as a result of phenotypic and/or genotypic drift [41]. Indeed, in our study, we note significant changes in community composition that occurred between the G_4_ and G_15_, which is not surprising given the movement between two different animal facilities and the multitude of factors concerned with animal husbandry known to influence the microbiome [42].

Moreover, we note that an additional aim of the G_4_ study was to simultaneously evaluate the role of host genetics for skin microbiota composition and susceptibility to autoimmune skin blistering, for which we induced an immunization-based disease model in a subset of the G_4_ mice [23]. This could in part explain, e.g., the significantly higher proportion of Firmicutes in the G_4_ compared to the G_15_ (ANOVA, *p* = 2.2 × 10^−16^), as this phylum is known to dominate the skin microbiota in the context of inflammatory disorders such as psoriasis and atopic dermatitis [43, 44]. However, we note that this difference can not be solely due to disease in the G_4_, as a subset of these animals was not immunized and Firmicutes are uniformly distributed across individuals (Fig. 3b).

**Fig. 6:**
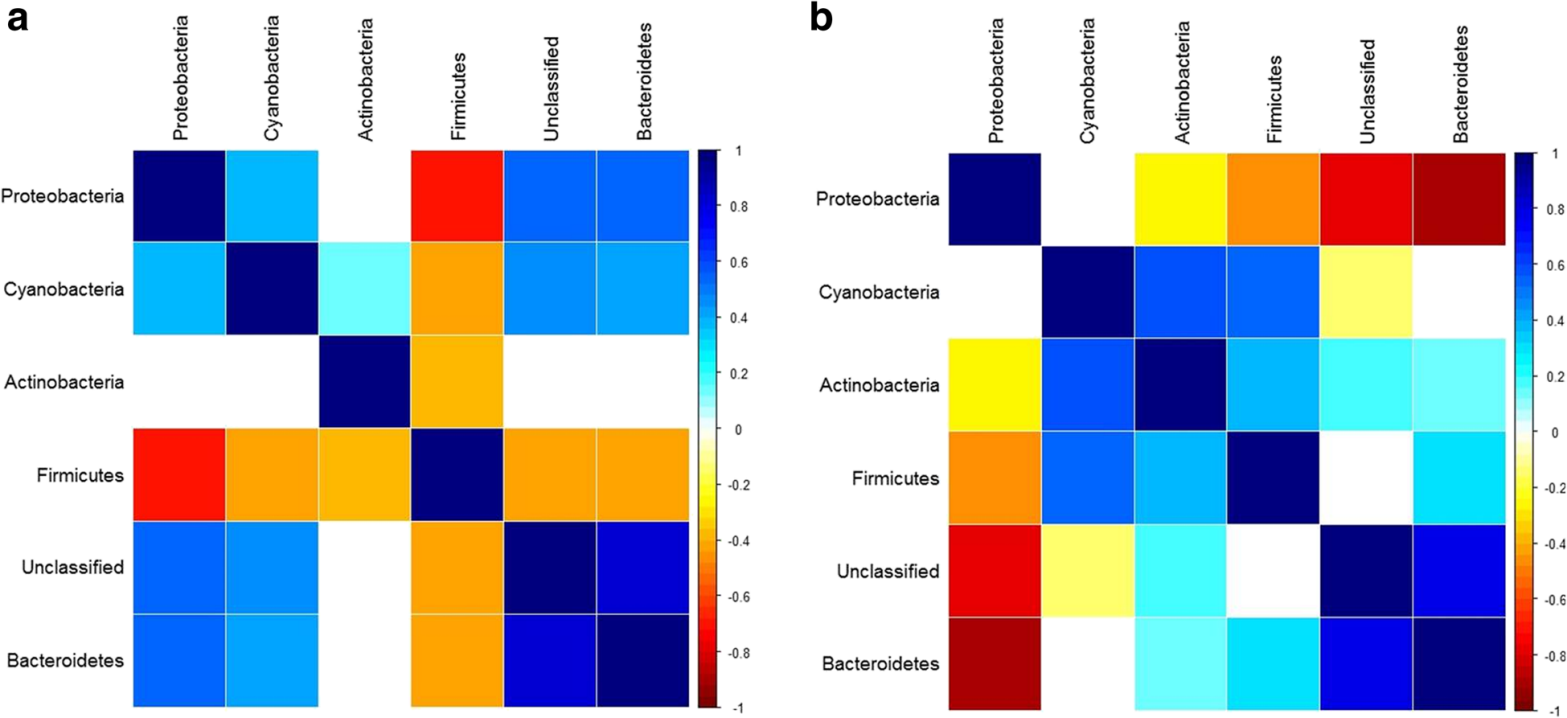
Correlations among CMM phyla in the standing communities of populations G_4_ and G_15_. **a** G_4_ population. **b** G_15_ population. Non-significant correlations (*p* > 0.05) after multiple testing correction are left blank

Finally, although our mapping approach treats each microbial taxon as an independent trait, microbial abundances may also change in an interrelated manner due to complex community interactions. To determine whether interdependencies between taxa may have changed in the context of the community-level differences we observe between the G_4_ and G_15_, we inspected the interactions between the relative abundances of CMM phyla within each population and indeed observe crucial changes in community interactions. In the G_4_ population, every phylum is strongly negatively correlated with Firmicutes, with Proteobacteria showing the strongest negative association (Fig. 6a). In the G_15_ population, we observe the opposite pattern; every phylum is negatively correlated with Proteobacteria, with Bacteroidetes exhibiting the strongest negative relationship (Fig. 6b). Thus, a change in the overall structure of community interactions between the two cohorts may also alter the nature of host genetic influence on the skin community, i.e., our observations may in part represent gene x environment interactions.

### 16S rRNA profiling at the DNA versus RNA level

Several possible explanations can be provided for the increased success in mapping 16S rRNA transcript-level traits. First, in comparison to the bacterial communities inhabiting the lower gastrointestinal tract, the skin harbors a lower biomass and is in constant contact with the environment. Thus, environmental noise from non-resident microbes, which are less likely to display active growth in the skin, is more likely to obscure the signal of resident microbes when 16S rRNA gene profiling is performed at the DNA level. As an example, we more closely examined the QTL analyses between DNA- and RNA-based abundances for unclassified Betaproteobacteria, a taxon for which the two relative abundance estimates are strongly and positively correlated (Spearman’s correlation: *r* = 0.62, *p* < 2.2 × 10^−16^). Additional file 15 shows the Manhattan plot for QTL mapping of unclassified Betaproteobacteria based on DNA and RNA. This reveals a strong overlap between the LOD scores generated for DNA- and RNA-based measurements, but the peak that defines a significant QTL on the RNA level does not reach the genome-wide significance threshold for DNA.

Second, in contrast to the example of the unclassified Betaproteobacteria QTL described above, we observe an overall poor correlation between the taxon abundances at the DNA versus RNA level, which in some cases is characterized by taxa with high activity but low abundance (see, e.g., unclassified Lachnospiraceae in Fig. 2). Thus, it is likely that many taxa observed at the RNA level are below the limits of reliable measurement at the DNA level. Further, observations at the bacterial transcript level may be more likely to be representative of interaction with the host. This hypothesis is consistent with previous observations of DNA- versus RNA-level bacterial community profiling in a dynamic aquatic habitat, where bacterial activity, but not presence alone, varied along with fluctuations in environmental parameters [45].

### Candidate genes and bacterial traits

In addition to the genomic intervals for which only single genes are present (*Cdh13* and *Nell2*, above), several other regions contain highly interesting candidate genes, whose potential functional role in host-microbe interactions is in some cases further supported by the bacterial traits with which they putatively interact. The QTL for *Acinetobacter_OTU51* (RNA level) on chromosome 1 contains a potential immune-related interaction, as the *Cops5* gene (synonyms *CSN5*, *Jab1*) found in this interval was shown to influence T cell development [46], whereas *Acinetobacter* itself was demonstrated to protect against allergic sensitization and inflammation in the skin by influencing the balance between TH1, TH2, and anti-inflammatory responses [47]. However, *Cops5* may alternatively or in addition represent another potential cancer-related interaction, as it is also known to play a critical role in cell proliferation, apoptosis, and regulation of genomic stability and DNA repair [48]. Abnormal expression of *Cops5* was demonstrated to impact carcinogenesis in several cancer types including breast cancer, laryngeal cancer, and oral squamous cell carcinoma (SCC) [49–51]. Further, Ivan et al. [52] assessed *Cops5* in various melanocytic lesions and found higher expression levels of *Cops5* in metastatic melanomas, suggesting that *Cops5* may influence the survival and growth of melanoma cells.

As mentioned above, the QTL for Deltaproteobacteria (DNA level) on chromosome 8 contains *Cdh13* (Fig. 5)*,* which is expressed in keratinocytes and involved in susceptibility to SCC and malignant melanoma. Interestingly, the Deltaproteobacteria class includes sulfate-reducing bacteria that generate hydrogen sulfide (H_2_S), which can have genotoxic properties [53] and is implicated in the pathogenesis of ulcerative colitis [54–56], a disease associated with increased colon cancer risk. On the other hand, H_2_S is also recognized as an endogenous gasotransmitter and was demonstrated to play a functional role in human cutaneous microvasculature [57] and a protective role in systemic sclerosis-associated skin fibrosis [58]. Thus, further functional characterization of potential H_2_S-producing bacteria in the skin and in the context of *Cdh13* as a regulator of keratinocyte proliferation and skin architecture may be warranted.

A final highly notable example is the QTL for *Halomonas* (RNA level) on chromosome 13, which contains the *Sox4* gene belonging to the *SoxC* class of transcription factors. *Sox4* is known to inhibit apoptosis and increase proliferation and is thus highly linked to carcinogenesis [59]. Foronda et al. [60] further addressed the role of *Sox4* (which is the only member of the *SoxC* class that is expressed in the skin) in skin homeostasis and cancer by making a skin-specific deletion of *Sox4* in combination with a chemically induced carcinogenesis model. The authors report reduced tumor progression and number in *Sox4*-deficient mice compared to wild type, indicating an oncogenic activity of *Sox4* in the skin. Interestingly, extracts from *Halomonas meridiana* bacteria isolated from brine pools of the Red Sea were demonstrated to have significant anticancer activity (apoptosis) in human cancer cell lines [61], raising the intriguing possibility that resident skin microbes play a role in endogenous anticancer activity from a hologenomic perspective.

## Conclusions

The path from QTL mapping to gene/mutation identification and functional characterization is complex and challenging. Through the use of high-resolution mapping and the introduction of phenotyping based on microbial activity, our study makes a substantial step towards understanding the host genetic component to interindividual variability in the skin microbiota and its potentially important fitness consequences. In particular, the preponderance of cancer-related candidate genes identified should motivate greater attention to the role of host-microbe interactions in cancer susceptibility and their potential as preventative and/or therapeutic targets. Finally, we suggest that adding activity-based community profiling may greatly enhance the capability to detect biologically meaningful host-microbe interactions in a wide variety of microbiome study settings.

## Methods

### Animals and skin sample collection

MRL/MpJ, NZM2410/J, BXD2/TyJ, and CAST/EiJ mice were purchased from the Jackson Laboratory (Maine, USA) and kept under conventional conditions. To generate a heterogeneous intercross line, these individuals were intercrossed at an equal strain and sex distribution as described previously [23]. Male and female offspring used in the study were transferred to separate cages according to family. Animals were held under specific pathogen-free conditions at a 12-h light/dark cycle with food and water ad libitum. All 270 animals (98 males and 172 females) were housed in the University of Lübeck, Germany, and sampled from the 15th generation of this advanced intercross line at a mean age of 5.9 months. All animal experiments were approved by the “Ministerium für Energiewende, Landwirtschaft, Umwelt und ländliche Räume des Landes Schleswig-Holstein” in Kiel, Germany (reference number: V 312–72241. 122–5 (12-2/09)).

An identical region from the left ear of each mouse was sampled, snap frozen, and stored at −80 °C until processing. During the dissection process, tools were carefully sterilized by flaming 70% ethanol. Total DNA and RNA were extracted simultaneously using the AllPrep DNA/RNA Qiagen kit. The working surface and pipettes were decontaminated with RNase AWAY® (Thermo Fisher Scientific). An additional 2-h room temperature incubation step was included after homogenization in order to increase the nucleic acids’ dissolution in the RLT buffer. RNA was treated with DNase (RNase-Free DNase Qiagen, stock solution concentration) for 15 min, twice. cDNA synthesis was performed using High-Capacity cDNA Reverse Transcription Kits (Applied Biosystems). In addition, RNA purity was checked by a negative reverse transcriptase (without transcriptase) PCR and agarose gel electrophoresis.

### 16S rRNA gene sequencing and processing

We amplified the V1–V2 regions of the bacterial 16S rRNA gene following a dual indexing approach for each sample. The primer pair (5′-*AATGATACGGCGACCACCGAGATCTACAC*XXXXXXXXTATGGTAATTGT *AGAGTTTGATCCTGGCTCAG*-3′) and (5′*CAAGCAGAAGACGGCATACGAGAT*XXXXXXXXAGTCAGTCAGCC *TGCTGCCTCCCGTAGGAGT*-3′) contains the Illumina P5 (forward) and P7 (reverse), denoted by *italics*, whereas the *underlined italic* sequences represent the broadly conserved bacterial primers 27F and 338R. A 12-base linker sequence (underlined only) was added to the bacterial primer in order to increase the annealing temperature of the sequencing primer, as recommended by Illumina. Both primers contained a unique eight-base multiplex identifier (Index; designated as XXXXXXXX) in order to tag each PCR product. PCR amplifications were conducted in a 12.5-μL volume containing 100 ng of either DNA or cDNA template using the Phusion® Hot Start II DNA High-Fidelity DNA Polymerase (Finnzymes, Espoo, Finland). Cycling conditions were as follows: initial denaturation for 30 s at 98 °C; 35 cycles of 9 s at 98 °C, 30 s at 55 °C, and 30 s at 72 °C for DNA, 30 cycles of 9 s at 98 °C, 30 s at 55 °C, and 30 s at 72 °C for cDNA; final extension for 10 min at 72 °C. Reactions were duplicated and products were merged in order to obtain a final volume of 25-μL PCR for each sample. Negative controls were performed using blank (template-free) reactions with different combinations of forward and reverse primers such that all primers were checked for contamination, which were required to be negative as inclusion criteria. Further, negative extraction controls for each round of DNA/RNA extraction (*n* = 14, i.e., in total, 28 negative extraction controls, as DNA and RNA are split during the procedure) were included. For all samples, PCR product concentrations were first quantified on an agarose gel using image analysis software (Bio-Rad). After quantification, products were mixed together to make equimolar subpools. Subpools were then extracted from agarose gel with the Qiagen MinElute Gel Extraction Kit and quantified with the Quant-iT™ dsDNA BR Assay Kit on a Qubit fluorometer (Invitrogen). Finally, subpools were combined in one equimolar pool for each library. Pools were further purified using AMPure® Beads (Agencourt), and complete libraries were run on an Agilent Bioanalyzer prior to sequencing, as recommended by Illumina. The Amplicon libraries were sequenced on a MiSeq using the MiSeq Reagent Kit v3 600 cycles sequencing chemistry.

No mismatch to the barcode was allowed while de-multiplexing (CASAVA, Illumina). Raw forward and reverse reads were merged in USEARCH (v.7) [62] as follows: forward and reverse reads were truncated before alignment at the first base where the quality score dropped below *Q* = 2; the maximum number of mismatches allowed in the overlap region was 2; the minimum length of the forward and reverse reads after truncating was 200 bp; the minimum length of the overlap region was 150 bp; the minimum length of the merged read was 270 bp; the maximum length of the merged read was 330 bp. Merged reads were filtered by the parameter of expected error (*E* = 0.5), as recommended [63]. Finally, chimeric sequences were removed in UCHIME [64] using the SILVA Gold reference database [65].

### Taxonomic classification and OTU binning

RDP Multi-Classifier (v.9.0) [66] implemented in Mothur (v.31) [67] was applied to assign taxonomy from the phylum to the genus level using a 0.80 confidence threshold and 1000 iterations. Sequences classified as Archaea, unknown, or Mitochondria were removed. Afterwards, sequences were aligned to the SILVA reference database. Sequences that failed to align were excluded. To avoid spurious OTUs, aligned sequences were further de-noised using the pre-cluster algorithm executed in Mothur, as recommended by Schloss et al. [68]. Prior to OTU binning, samples were rarefied to an even sequence depth of 3500, except for one sample that had 3134 reads, in order to control for varied sequencing depth and biases in ecological community analysis. Operational taxonomic units were binned at a 97% similarity threshold using the Mothur average clustering algorithm.

### SNP genotyping and founder haplotype reconstruction

Genomic DNA was isolated from liver tissue and incubated in 500 μL of 50 mM NaOH at 95 °C for 2 h. The reaction was neutralized by the posterior addition of 50 μL of 1 M Tris-HCl (pH 8.0). DNA was further processed with DNeasy Blood & Tissue Kit (Qiagen) according to the manufacturer’s instructions. The extracted DNA was quantified using NanoDrop and normalized to 50 ng/μL in TE buffer (10 mM Tris, 1 mM EDTA; pH = 8). We used the high-density mouse universal genotyping array, MegaMuga (Illumina), which can hybridize up to 77,800 SNPs, to genotype the mice as well as the four founder individuals. Raw genotyping data were first analyzed using GenomeStudio Data Analysis Software (Illumina). Then, using PLINK (v.1.07) [69], SNPs missing in the founder individuals and SNPs with a minor allele frequency of 10% or below were removed. We obtained 53,203 informative SNP markers distributed genome-wide, with an average spacing of 0.04 Mb and standard deviation of 0.09 Mb. The four founder haplotype probabilities were calculated at each SNP marker for every sample by splitting the chromosomes using the “*hdesign*” function from the “HAPPY” (v.2.4) R package [70].

### Core Measurable Microbiota

To determine the set of microbial traits to be included in the QTL mapping, we defined a Core Measurable Microbiota (CMM) to include only bacterial taxa (genus to phylum level) with at least five reads in at least half of the samples, and species-level OTUs with at least five reads in at least one third of the samples. These thresholds were applied to the DNA- and RNA-based datasets separately, whereby in total 92 taxa (genus to phylum level) and 44 OTUs were included. We added a value of 0.5 to the absolute abundances of all CMMs, and then converted the absolute abundances into relative abundances. In order to reduce skewness, relative abundances were log_10_-transformed.

### SourceTracker analysis

SourceTracker [26] analysis was performed on the level of mapped genera and species-level OTUs (97% similarity threshold) under default parameters. The training set included the negative extraction controls for which a weak PCR product was detectable and sufficient post quality filtering read number was achieved (16 out of 28 with >1000 reads, remaining samples containing 1 to 797 (mean 99) reads or no PCR product).

### Summary statistics of the CMMs and partitioning of variance

All statistical analyses were performed in R (v.3.2.2) [71]. In order to assess the magnitude of variability of the CMMs, summary statistics were calculated on each CMM (taxa and OTUs). Multivariate analysis of variance was applied on the CMMs log_10_-transformed relative abundances. We tested the effects of weight, gender, age, and cage environment on the variation in the CMM relative abundances in DNA- and RNA-based datasets; the three latter factors showed a significant overall effect (*p* ≤ 0.05) and were thus included in the QTL mapping model. To measure the effects of these factors on variation in CMM trait abundances across samples, we built a mixed effects model in the “lme4” (v.1.1-10) R package [72] for each CMM trait (taxa and OTUs) in the DNA- and RNA-based datasets, which included gender and age as fixed factors and cage as a random term. The variance components were measured using the “VarCorr” function. Marginal *R* ^2^, which represents the proportion of variance explained by fixed factors [73], was calculated using the “r.squaredGLMM” function in the “MuMIn” (v.1.15.1) R package [74].

### QTL mapping

Linkage mapping was performed with a mixed model approach using the “QTLRel” (v. 0.2-14) R package [75] in DOQTL (v. 1.2.0) [76]. We fit an additive model that regresses the log_10_-transformed relative abundances of each CMM trait (taxa and OTUs) on the four founder haplotype contributions. To adjust for different degrees of mice relatedness, a kinship matrix was defined by calculating correlation coefficients between samples using the “*kinship.probs*” function from the DOQTL package. The kinship matrix was incorporated into the model instead of pedigree records, as recommended by Svenson et al. [77] and Gatti et al. [76]. Gender and age (expressed in days) were incorporated as fixed covariates and cage as a random term in the mapping model. To determine significance thresholds for the LOD scores of each marker/trait, we applied a permutation procedure described by Churchill and Doerge [78]. This procedure consists of shuffling the phenotypes (i.e., the log_10_ relative abundances) across the genotypes and re-runs the QTL model to generate new LOD scores. We repeated this process 5000 times for each marker/trait. Afterwards, using the newly generated distribution of LOD scores, we determined the 90th and 95th percentiles and used them to define the suggestive (0.1) and significant thresholds, respectively. Finally, we compared the LOD scores generated in the original QTL scan to the defined significance thresholds: suggestive (genome-wide alpha ≤ 0.1) and significant (genome-wide alpha ≤ 0.05). Only the LOD scores that met or exceeded either the suggestive or significant thresholds were considered to indicate the presence of a putative QTL and reported in our results. We set QTL confidence intervals at 1.5 LOD drops on either side of the peak position.

### Quantitative and ecological analysis

Alpha diversity indices (i.e., Shannon entropy and Chao1) were calculated in the “vegan” (v.2.3.0) R package [79] on the entire dataset at both the genus and OTU levels. For each alpha diversity measure, we calculated summary statistics and partitioning of variance and performed QTL mapping only for Chao1, as described above. Comparison of mean relative abundances of major phyla and genera between the standing and active communities was performed using a paired Wilcoxon test. The correlation of relative abundances between the standing and active dataset was performed using Spearman’s correlation. Correlations between the abundances of major phyla in the standing communities of generations 4 and 15 were calculated using Spearman’s correlation in the “Hmisc” (v.3.17-0) R package [80]. *p* values were adjusted using the Benjamini and Hochberg method [27], and correlation coefficients were visualized using the “corrplot” (v.0.73) R package [81].

### Real-time quantification of bacterial traits

16S rRNA gene primers targeting Betaproteobacteria, Epsilonproteobacteria, *Streptococcus*, and total bacteria were used to perform real-time quantification on a random subset of 80 out of the 270 mice. The taxon-specific primers include F_AACGCGAAAAACCTTACCTACC and R_TGCCCTTTCGTAGCAACTAGTG for Betaproteobacteria, F_TAGGCTTGACATTGATAGAATC and R_CTTACGAAGGCAGTCTCCTTA for Epsilonproteobaceria [82], F_CTWACCAGAAAGGGACGGCT and R_AAGGRYCYAACACCTAGC for *Streptococcus* [83], and F_ACTCCTACGGGAGGCAGCAG and R_ATTACCGCGGCTGCTGG for total bacteria [84]. Real-time quantitative PCR was carried out in a volume of 10 μL on a PikoReal Real-Time PCR System using 96-well plates and three technical replicates for each sample. Each PCR mixture consisted of 5 μL of PowerUp SYBR PCR Master Mix (Applied Biosystems), 0.5 μL of each primer (10 μM), 2.5 μL of water, and 2 μL of the original cDNA template used for 16S rRNA gene sequencing (1:20 dilution). The amplification program consisted of (i) initial step at 95 °C for 10 min, (ii) 45 cycles of denaturation at 95 °C for 15 s and annealing/extension at 60 °C for 1 min, and (iii) 1 cycle at 60 °C for 30 s and a melt ramp from 60 to 95 °C. The relative quantification of a given bacterial trait was determined by comparing taxon-specific and total bacteria *C*_T_ values as expressed ${2}^{-\left(\mathrm{delta}\hbox{-} \mathrm{delta}\ {C}_{\mathrm{T}}\ \mathrm{values}\right)}$.

### Pathway and gene ontology enrichment analysis

For the enrichment analysis, we selected a list of genes that includes the two nearest genes to the peak SNP on either side of the chromosome (maximum four genes per interval) and performed the analyses based on two categories including pathways (Biocarta) and ontologies (Biological Processes). We used the tool Enrichr [85] and sorted the results based on the combined score from the enrichment analysis.

## Additional files

Additional file 1: Figure S1. Principal coordinate analysis of beta diversity indices of skin microbiota in populations G_15_ and G_4_. (A) Bray-Curtis, (B) Jaccard indices. Indices are calculated on genera abundances after normalization of sequencing depth to 2500 reads per sample in both populations G_15_ and G_4_. DNA: standing, RNA: active. Goodness of fit: Bray-Curtis, *r* ^2^ = 0.38, *p* = 1.10^−5^; Jaccard, *r* ^2^ = 0.47, *p* = 1.10^−5^, based on 1000 permutations. SD: standard deviation. (TIF 392 kb)

Additional file 2: Table S1. Summary statistics of CMM taxa and alpha diversity indices as profiled based on DNA. Means, standard deviations (STD), minimum (Min), maximum (Max), and coefficient of variation (CV) values of relative abundances in population G_15_ (*n* = 270). When taxa are redundant, only the lowest rank is shown. Un: unclassified. (XLSX 15 kb)

Additional file 3: Table S2. Summary statistics of CMM taxa and alpha diversity indices as profiled based on RNA. Means, standard deviations (STD), minimum (Min), maximum (Max), and coefficient of variation (CV) values relative abundances in population G_15_ (*n* = 270). When taxa are redundant, only the lowest rank is shown. Un: unclassified. (XLSX 15 kb)

Additional file 4: Table S3. Summary statistics of CMM OTUs and alpha diversity indices as profiled based on DNA. Means, standard deviations (STD), minimum (Min), maximum (Max), and coefficient of variation (CV) values of relative abundances in population G_15_ (*n* = 270). Un: unclassified. (XLSX 12 kb)

Additional file 5: Table S4. Summary statistics of CMM OTUs and alpha diversity indices as profiled based on RNA. Means, standard deviations (STD), minimum (Min), maximum (Max), and coefficient of variation (CV) values of relative abundances in population G_15_ (*n* = 270). Un: unclassified. (XLSX 12 kb)

Additional file 6: Table S5. Estimates of proportions of explained variance by explanatory variables for genus- to phylum-level taxa (DNA). Estimates for random components (cage and residual) and fixed terms (gender and age) are calculated for each of the CMM taxa log_10_-relative abundances and alpha diversity indices as profiled based on DNA in population G_15_ (*n* = 270). When taxa are redundant, only the lowest rank is shown. Un: unclassified. (XLSX 14 kb)

Additional file 7: Table S6. Estimates of proportions of explained variance by explanatory variables for genus- to phylum-level taxa (RNA). Estimates for random components (cage and residual) and fixed terms (gender and age) are calculated for each of the CMM taxa log_10_-relative abundances and alpha diversity indices as profiled based on RNA in G_15_ (*n* = 270). When taxa are redundant, only the lowest rank is shown. Un: unclassified. (XLSX 14 kb)

Additional file 8: Table S7. Estimates of proportions of explained variance by explanatory variables for OTUs (DNA). Estimates for random components (cage and residual) and fixed terms (gender and age) are calculated for each of the CMM OTUs log_10_-relative abundances and alpha diversity indices as profiled based on DNA in G_15_ (*n* = 270). When taxa are redundant, only the lowest rank is shown. Un: unclassified. (XLSX 13 kb)

Additional file 9: Table S8. Estimates of proportions of explained variance by explanatory variables for OTUs (RNA). Estimates of proportions of explained variance by random components (cage and residual) and fixed terms (gender and age) are calculated for each of the CMM OTUs log_10_-relative abundances and alpha diversity indices as profiled based on RNA in G_15_ (*n* = 270). When taxa are redundant, only the lowest rank is shown. Un: unclassified. (XLSX 13 kb)

Additional file 10: Table S9. Residuals of the linear mixed models for all mapped taxa and alpha diversity measures. Un: unclassified. (XLSX 1069 kb)

Additional file 11: Figure S2. Manhattan plot of *Neisseria_OTU1320* QTL mapping. Significant thresholds (*p* ≤ 0.05) are shown in a continuous line; suggestive thresholds (*p* ≤ 0.10) are shown in a discontinuous line. Chr: chromosome. (TIF 219 kb)

Additional file 12: Table S10. Functions of the potential candidate genes. Un: unclassified. (XLSX 15 kb)

Additional file 13: Figure S3. Pathway and biological process enrichment analysis of candidate regions. A. Pathways analysis (Biocarta). B. Biological processes. Significant enrichment results after correction for multiple testing (*p* ≤ 0.1) are displayed in color; non-significant enriched terms are colorless. For the biological process analysis, only combined scores higher than 2.5 are reported. (TIF 581 kb)

Additional file 14: Table S11. Full details of pathway and biological process enrichment analyses for candidate regions. (XLSX 71 kb)

Additional file 15: Figure S4. Manhattan plot for unclassified Betaproteobacteria QTL. Significant thresholds (*p* ≤ 0.05) are shown in a continuous line; suggestive thresholds (*p* ≤ 0.1) are shown in a discontinuous line. Chr: chromosome, Un: unclassified. (TIF 572 kb)

## Abbreviation

OTU: Operational taxonomic unit
