# Supplementary figures and images for: Improved detection of gene-microbe interactions in the mouse skin microbiota using high-resolution QTL mapping of 16S rRNA transcripts
Source: Microbiome. 2017 Jun 6;5:59. doi: 10.1186/s40168-017-0275-5 (PMC5461731; doi:10.1186/s40168-017-0275-5)

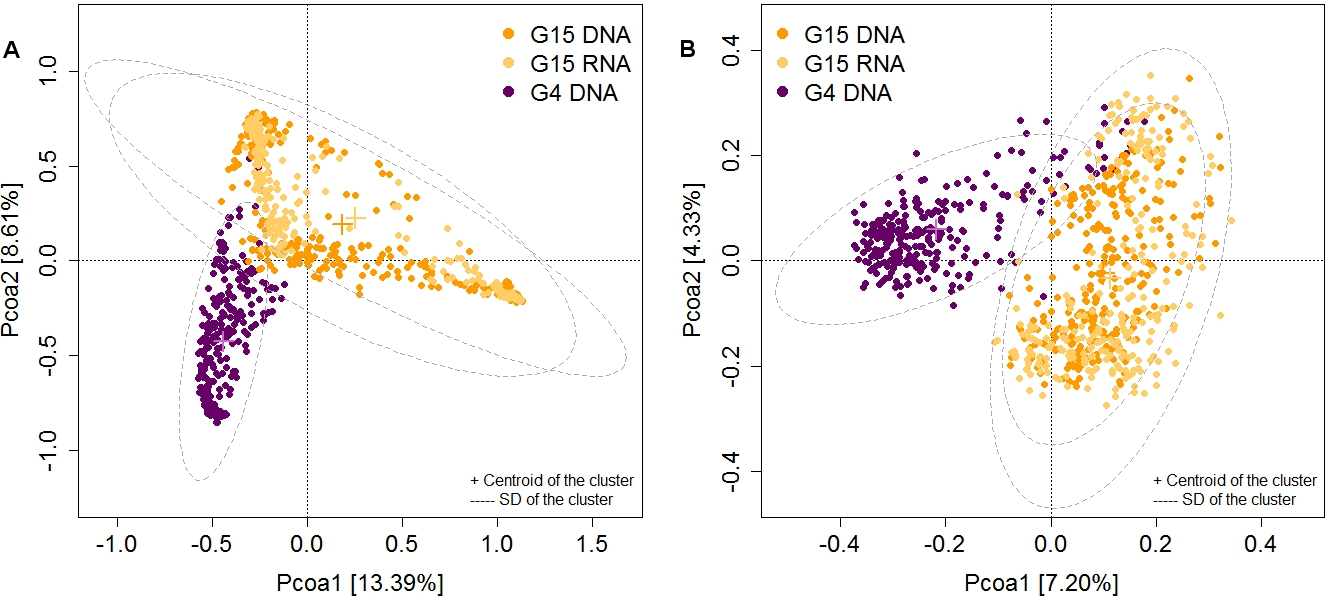

Supplement: Supplementary file 1 — Principal coordinate analysis of beta diversity indices of skin microbiota in populations G15 and G4. (A) Bray-Curtis, (B) Jaccard indices. Indices are calculated on genera abundances after normalization of sequencing depth to 2500 reads per sample in both populations G15 and G4. DNA: standing, RNA: active. Goodness of fit: Bray-Curtis, r 2 = 0.38, p = 1.10−5; Jaccard, r 2 = 0.47, p = 1.10−5, based on 1000 permutations. SD: standard deviation. (TIF 392 kb) [file 40168_2017_275_MOESM1_ESM.tif]

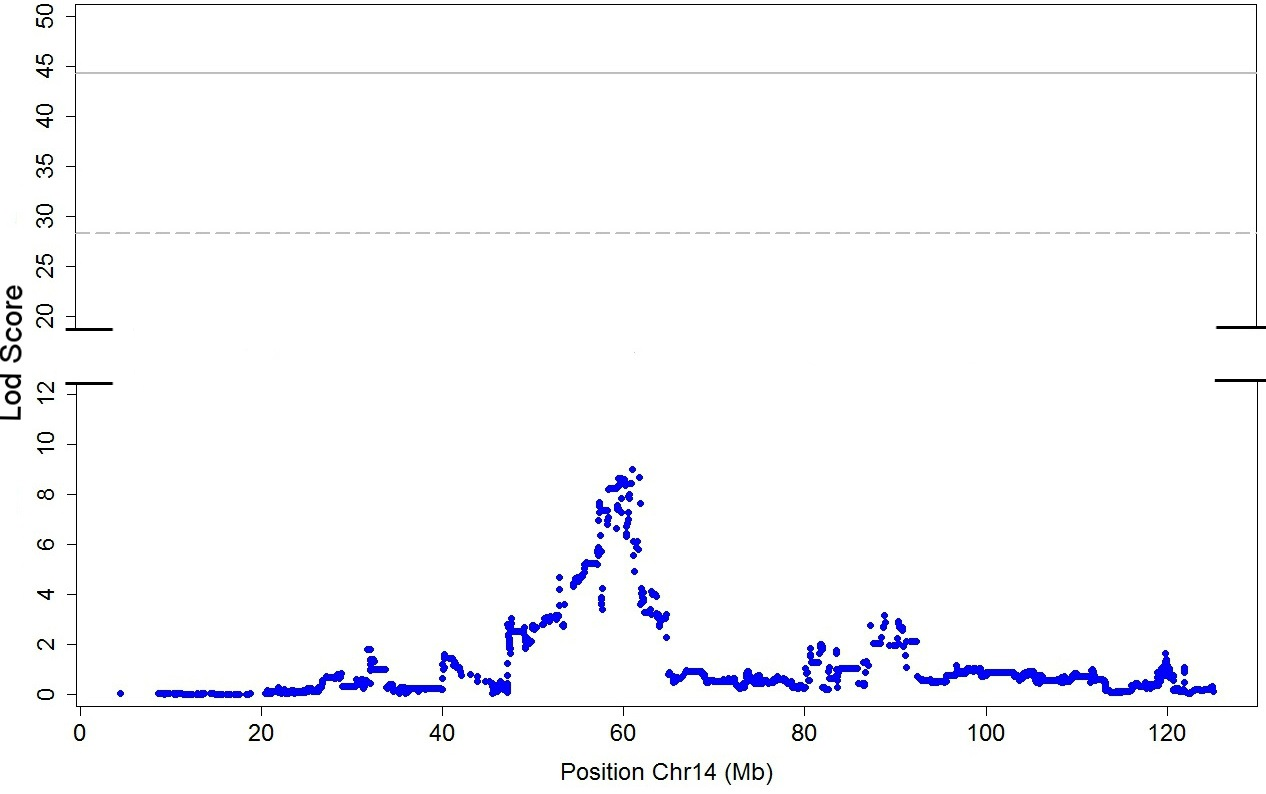

Supplement: Supplementary file 11 — Manhattan plot of Neisseria_OTU1320 QTL mapping. Significant thresholds (p ≤ 0.05) are shown in a continuous line; suggestive thresholds (p ≤ 0.10) are shown in a discontinuous line. Chr: chromosome. (TIF 219 kb) [file 40168_2017_275_MOESM11_ESM.tif]

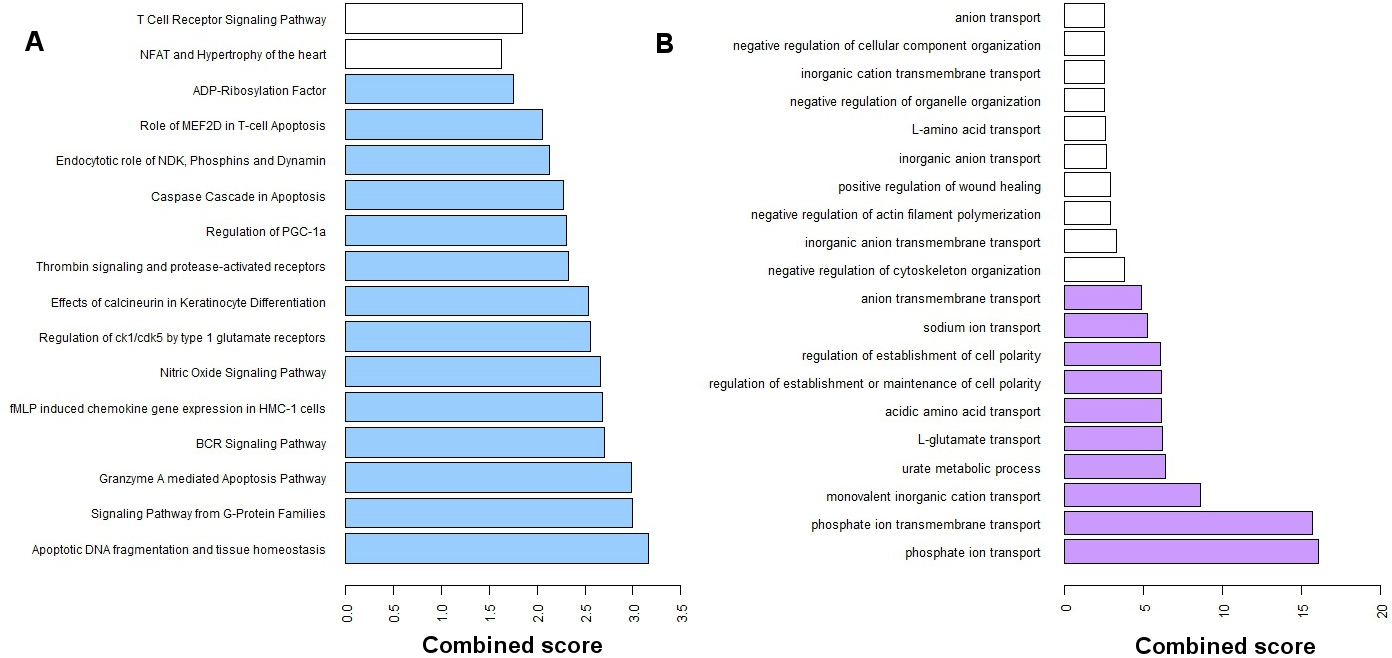

Supplement: Supplementary file 13 — Pathway and biological process enrichment analysis of candidate regions. A. Pathways analysis (Biocarta). B. Biological processes. Significant enrichment results after correction for multiple testing (p ≤ 0.1) are displayed in color; non-significant enriched terms are colorless. For the biological process analysis, only combined scores higher than 2.5 are reported. (TIF 581 kb) [file 40168_2017_275_MOESM13_ESM.tif]

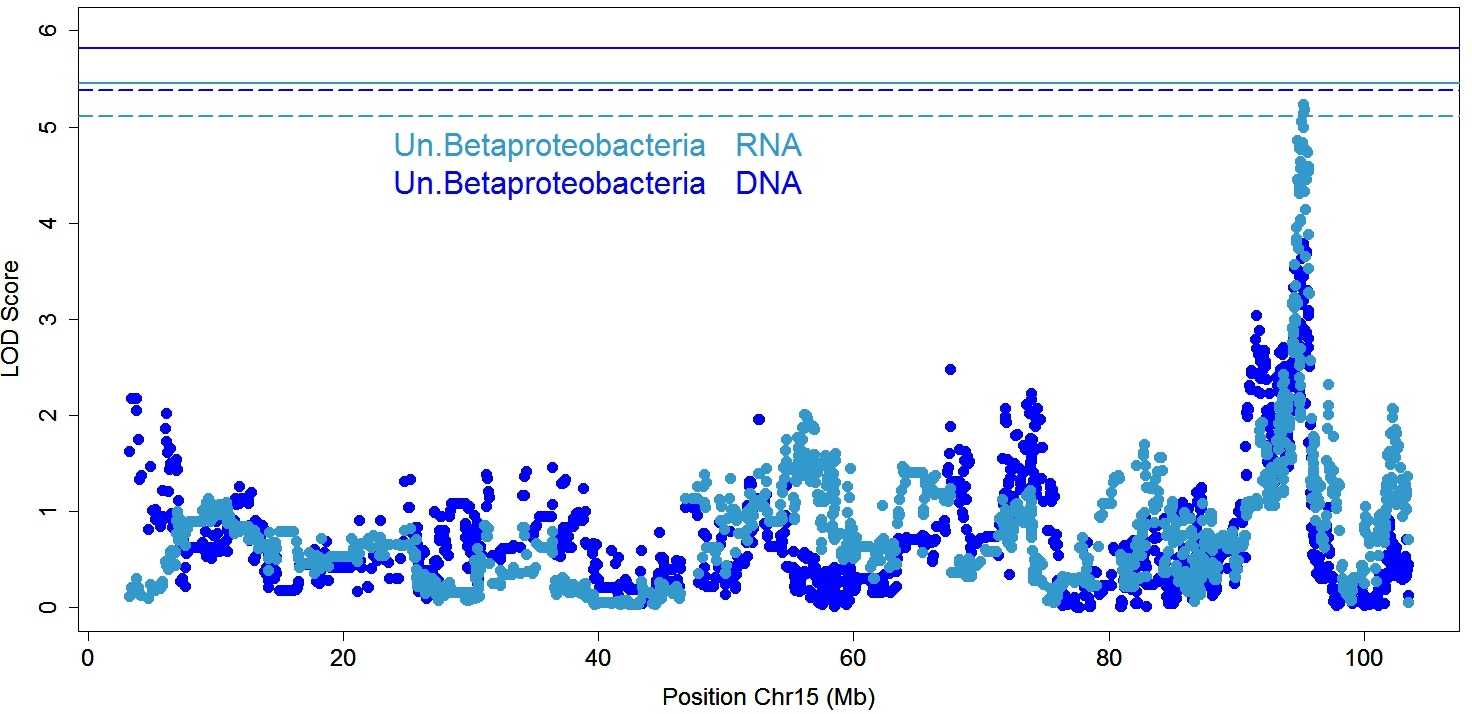

Supplement: Supplementary file 15 — Manhattan plot for unclassified Betaproteobacteria QTL. Significant thresholds (p ≤ 0.05) are shown in a continuous line; suggestive thresholds (p ≤ 0.1) are shown in a discontinuous line. Chr: chromosome, Un: unclassified. (TIF 572 kb) [file 40168_2017_275_MOESM15_ESM.tif]
